# Histone methyltransferase KMT2A: Developmental regulation to oncogenic transformation

**DOI:** 10.1016/j.jbc.2024.107791

**Published:** 2024-09-18

**Authors:** Jayme Ogino, Yali Dou

**Affiliations:** 1 Division of Pediatric Hematology-Oncology, Children’s Hospital Los Angeles, Los Angeles, California, USA; 2 Department of Medicine, Norris Comprehensive Cancer Center, University of Southern California, Los Angeles, California, USA

**Keywords:** epigenetics, histone methylation, leukemia, cancer therapy, development, MLL, KMT2A

## Abstract

Our current understanding of epigenetic regulation is deeply rooted in the founding contributions of Dr C. David Allis. In 2002, Allis and colleagues first characterized the lysine methyltransferase activity of the mammalian KMT2A (MLL1), a paradigm-shifting discovery that brings epigenetic dysregulation into focus for many human diseases that carry KMT2A mutations. This review will discuss the current understanding of the multifaceted roles of KMT2A in development and disease, which has paved the way for innovative and upcoming approaches to cancer therapy.

Eukaryotic gene expression is tightly regulated by a concerted series of histone and DNA modifications, generally referred to as epigenetic modifications. Aberrant activity in epigenetic modifications has been linked to a diverse and growing list of human diseases. Our current understanding of epigenetic regulation is deeply rooted in the founding contributions of Dr C. David Allis. Notably, his landmark discoveries have shed light on the intricate epigenetic mechanisms underlying normal developmental processes as well as the pathogenesis of human diseases, including malignancies. In this review, we will focus on the founding member of the KMT2 (lysine methyltransferase 2) family enzymes, KMT2A, which was initially reported by Allis and colleagues as a histone three lysine 4 (H3K4) methyltransferase (1). KMT2A has garnered significant attention in the scientific and medical community due to its essential role in transcription regulation, and high mutation rate in a broad spectrum of human diseases. This review provides a comprehensive analysis of KMT2A (MLL1), encompassing its structure, function, and role in development and disease. Drawing upon recent research findings and clinical insights, this review additionally offers an in-depth understanding of the promising therapeutic strategies in KMT2A-mediated malignancies.

## Structure, function, and regulation

The lysine methyltransferase family 2 (KMT2) family, commonly known as MLL (Mixed Lineage Leukemia), comprises a highly conserved group of histone methyltransferases that are responsible for mono-, di-, and tri-methylation at histone three lysine 4 (H3K4me1/2/3, respectively) through their conserved SET domain (Su(var)3–9, Enhancer of zester and Trithorax) (2, 3, 4). In lower eukaryotes, there is usually only one KMT2 gene, *e.g. Set1* in yeast and *Trithorax 1* (*Trx1*) in *Tetrahymena thermophila*. In *Drosophila*, there are three KMT2 genes, *Trx*, *Trx-related* (Trr), and *dSet1*. Gene duplications during mammalian evolution led to two paralogs in each gene group: Trx-related *KMT2A (MLL1*) and *KMT2B (MLL2)*, Trr-related *KMT2C (MLL3)* and *KMT2D (MLL4)*, and dSET1-related *KMT2F (SET1A)* and *KMT2G (SET1B)* (3). The increasing complexity of the KMT2 family correlates with the diversity and adaptability of organisms, suggesting potential sub-functionalization and regulatory complexity of *KMT2* genes (5).

**Figure 1:**
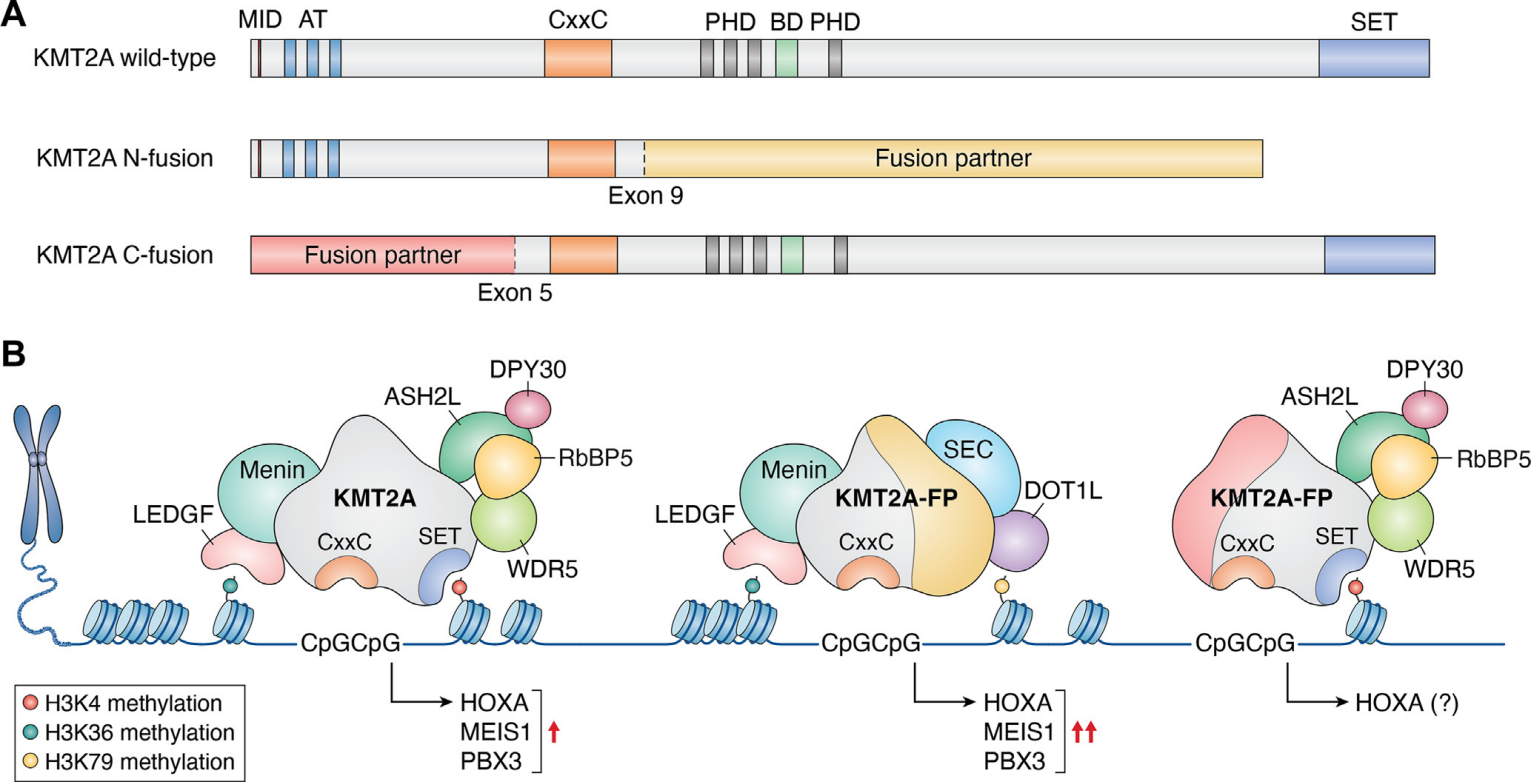
**KMT2A and KMT2A fusion proteins.***A*, schematics of the domain structures in KMT2A and KMT2A fusion proteins. The PHD and SET domains are preserved in KMT2A C-fusion proteins, which are observed in sarcomas. KMT2A N-fusion proteins, frequently seen in hematologic malignancies, loose the PHD and SET domains. See text for details. *B*, KMT2A and KMT2A fusion proteins regulate multiple steps in transcription activation through exerting or recruiting different chromatin remodeling activities at developmentally important genes. KMT2A N-fusion proteins result in the upregulation of the posterior *HOXA genes, and homeobox genes MEIS1 and PBX3,* driving leukemogenesis.

Compared to other KMT2 enzymes, KMT2A is highly homologous to KMT2B, sharing the MENIN (encoded by multiple endocrine neoplasia type 1 (MEN1))-interaction domain, N-terminal AT-hooks, and a CxxC domain. These domains are unique to KMT2A/B, responsible for their chromatin recruitment to MENIN-binding regions, AT-rich DNA, and unmethylated CpG islands (CGI), respectively (6). The C-terminal catalytic SET domain of KMT2A confers mono-, di- and tri-methylation on histone H3K4 (H3K4me1/2/3). More recently, we have reported that KMT2A can methylate non-canonical substrate Borealin (5, 7). Between the CxxC and SET domains, there are four PHD (plant homeotic domain) domains in KMT2A that regulate KMT2A stability, activity, and intra-protein interactions (8, 9, 10). KMT2A activity and functions are also subject to tight regulation by its interacting partners. For example, the catalytic activity of KMT2A is drastically enhanced by interactions with the WD repeat protein 5 (WDR5), retinoblastoma binding protein 5 (RbBP5), ASH2L, and DPY30 (11, 12). These proteins maintain the integrity of the KMT2A complex and are essential in dictating how KMT2A interacts with its substrate within the nucleosome core particles (Fig. 1) (11, 13, 14, 15, 16).

The functional repertoire of KMT2A and its cognate H3K4me have been well described in transcriptional regulation (5, 17, 18, 19). KMT2A is highly enriched at both gene promoters and enhancers, promoting transcription activation *via* H3K4me reader proteins (20). H3K4me also acts with other histone modifications to fine-tune the chromatin states and transcription outcomes (21, 22). Recent studies have broadened the role of KMT2A in transcription regulation through its downstream targets. Specifically, long-noncoding RNAs transcribed from *HOX* gene clusters, recruit KMT2A to specific genomic loci to regulate cell self-renewal and hematopoiesis (23). Beyond transcription regulation, KMT2A plays an important role in cell cycle regulation and genome stability (7, 24, 25). Given these multifaceted functions, KMT2A aberrations, including both genetic alterations and epigenetic dysregulation, often drive the pathogenesis of diverse human disorders, including neurodevelopmental syndromes and a broad range of cancers (26, 27).

## KMT2A and human developmental syndromes

KMT2A is essential for embryonic development. Early murine models have shown that heterozygous deletion of *KMT2A* is associated with defects in the axial skeleton patterns and hematopoiesis, whereas homozygous mutations in *KMT2A* result in embryonic lethality (6, 28). Post-natal and adult mice with KMT2A deficiency lead to changes in gene expression patterns as it relates to neuronal plasticity, with impaired cognition and increased anxiety (29, 30), supporting a causal role for KMT2A loss of function in a spectrum of neurodevelopmental syndromes.

*KMT2A* germline mutations have a well-established association with Wiedemann-Steiner Syndrome (WDSTS, OMIM#605130). This autosomal dominant syndrome is characterized by intellectual disability, developmental delay, behavioral difficulties, hypotonia, feeding difficulties, and a myriad of structural abnormalities (short stature, hypertrichosis, thick eyebrows, long eyelashes, narrow and down slanting palpebral fissures, broad nasal tip, clino-, or brachydactyly) (31). *KMT2A* mutations have also been identified in individuals initially clinically diagnosed with Coffin-Siris syndromes (CSS1, OMIM #135900; CSS2, OMIM #614607; CSS3, OMIM #614608; CSS4, OMIM #614609; CSS5, OMIM #616938; CSS6, OMIM #617808; CSS7, OMIM #618027; CSS8, OMIM #618362; CSS9, OMIM #615866; CSS10, OMIM #618506; CSS11, OMIM #618779; CSS12, OMIM #619325), Nicolaides-Baraitser syndrome (OMIM #601358), Kabuki syndrome (KS1, OMIM #147920; KS2, OMIM #300867), Cornelia De Lange syndromes (CdLS1, OMIM #122470; CdLS2, OMIM #300590; CdLS3, OMIM #610759; CdLS4, OMIM #614701; CdLS5, OMIM #300882), and Rubinstein-Taybi syndrome (RSTS1, OMIM #180849; RSTS2; OMIM #613684) (32). In such cases, features not traditionally associated with WDSTS were also identified, including vision problems, genitourinary problems, and microcephaly (31, 32). With the expanding accessibility of whole exome sequencing, the clinical heterogeneity associated with *KMT2A* mutations continues to expand, most recently including a spectrum of cardiac defects, dental anomalies, endocrinopathies, immune dysfunction, and eosinophilia (32, 33).

As is the case for many syndromes with a neurodevelopmental component, current management is supportive of behavioral therapy and neuropsychiatric medications, with no available targeted therapeutic options. Histone deacetylase (HDAC) inhibitors have shown preclinical promise in addressing the structural and functional neurologic defects in Kabuki Syndrome and Say-Barber-Biesecker-Young-Simpson Syndrome (OMIM# 603736), both driven by mutations in chromatin modifier genes KMT2D/KDM6A and KAT6B, respectively (34, 35). These findings hint at the therapeutic potential of targeting epigenetic regulators in neurodevelopmental disease, including those with underlying *KMT2A* mutations. Along similar lines, preclinical models show that activities of KMT2A and the opposing H3K4 demethylase KDM5A (Lysine-specific demethylase 5A), a histone lysine demethylase mutated in several neurodevelopmental disorders (36, 37), are tightly balanced such that the behavioral and structural effects due to the depletion of either are restored to a degree by depleting their respective counterpart (38). The delicate balance of KMT2A and KDM5A may be a therapeutic strategy warranting further exploration.

## KMT2A and hematologic malignancies

*KMT2A* aberration is a key feature across a spectrum of malignancies (5, 27). Structural variations in *KMT2A* are well-known cytogenetic features in hematological malignancy, accompanied by a characteristically aggressive nature, poor response to chemotherapy, and a high rate of relapse. *KMT2A* partial tandem duplications (PTD), most commonly involving exons 2 to 9, are observed in 10% of myelodysplastic syndrome/acute myeloid leukemia (MDS/AML) cases, and are associated with disease progression and relapse (39, 40). Similarly, individuals with leukemias harboring *KMT2A*-amplification (KMT2A-a; four or more copies of the *KMT2A* gene) have dismal outcomes, with median survival reported to be 2 to 3 months from the time of diagnosis (41). Limited clinical and cytogenetic information is known about KMT2A-a leukemias; these malignancies are associated with prior exposure to chemotherapy, older age, complex karyotype, and *TP53* mutations (41, 42, 43). However, the cytogenetic events that lead to KMT2A-a and the molecular consequences of KMT2A-a remain to be explored.

The most frequent structural variation of *KMT2A* in AML and acute lymphoblastic leukemia (ALL) is *KMT2A*-rearrangement (KMT2A-r), which is present in 10% of hematologic malignancies. There are over 90 unique oncogenic fusion partners documented to date (44). The most common fusion partner, *AFF1* (*AF4*), followed by *MLLT3* (*AF9*), *MLLT1* (*ENL*), and *MLLT10* (*AF10*), account for a large majority of observed cases of KMT2A-r leukemias (44). Analysis of *KMT2A* breakpoint distributions shows that most breakpoints occur distal to exon 9, thus the AT-hook and CxxC domains are preserved, but the PHD and SET domains are lost (Fig. 1) (44, 45). By preserving the MENIN and DNA interaction domains, KMT2A fusion proteins are recruited to the canonical KMT2A wild-type (WT) targets, such as posterior *HOX* genes, in KMT2A-r leukemias (1). KMT2A fusion proteins also exhibit promiscuous behavior by binding to genes that are not normally regulated by KMT2A. The KMT2A fusion targets vary among leukemia lineage subtypes (46). KMT2A fusion proteins can recruit the histone three lysine 79 (H3K79) methyltransferase, DOT1L, or core members of the super elongation complex *via* the C-terminal fusion partners (47, 48), which in turn enhance transcription elongation and constitutive transactivation of genes related to anti-apoptosis and/or differentiation block (48, 49). Recent evidence also suggests a role for KMT2A fusion proteins at distal enhancers to promote transcription activation *via* enhancer-promoter interactions (50). This mechanism results in the aberrant overexpression of canonical KMT2A targets such as the posterior *HOXA* genes*,* and homeobox genes *MEIS1* and *PBX3,* the hallmark gene expression pattern found in many KMT2A-r leukemias. Higher expression of these genes are associated with worse overall survival (51, 52, 53).

Strikingly, KMT2A-r is observed in nearly 80% of cases of infant B-cell acute lymphoblastic leukemia (B-ALL) (54). In such cases, KMT2A-r has been observed to be a prenatal event often occurring in the pre-hematopoietic, pluripotent cells (55). When genetic aberrations occur in genes that govern cell plasticity and differentiation, namely *KMT2A*, the resultant leukemia cell may have complex immunophenotypes and transcriptional states resembling the earliest precursor cells. In support of this, the transcriptional features of early lymphocyte precursors have been uniquely linked to KMT2A-r infant leukemia in comparison to other childhood ALLs (56). This feature emphasizes the early acquisition of the *KMT2A* aberration and may underlie the 50% mortality rate observed in infant KMT2A-r BALL (56, 57). Other studies have shown that KMT2A-r leukemia can maintain myeloid and lymphoid immunophenotype simultaneously or sequentially through lineage switch, offering an explanation for the poor response to standard ALL and AML chemotherapy regimens in comparison to those with wild-type *KMT2A* (58).

Across all ages, KMT2A-r is a predictive biomarker of unfavorable treatment response and poor outcomes for patients with ALL and AML. For the majority, predicted survival outcomes are less than 40%, lagging behind individuals without KMT2A-r (57). While the age of the patient and the lineage presentation of the leukemia impact treatment outcomes, the probability of disease relapse on current treatment regimens ranges from a sobering 12 to 60% (54, 59). Following relapse, the likelihood of achieving remission, let alone a clinical response, exponentially decreases with each relapse. Standard chemotherapeutic agents utilized to treat KMT2A-r ALL and AML work through a variety of mechanisms including DNA alkylation, interference with microtubule polymerization, or inhibition of topoisomerase II activity (60). The patient under chemotherapeutic treatment is burdened with off-target effects that can cause delays in treatment as well as short- and long-term health complications (60). As chemotherapeutic options are exhausted, chimeric antigen receptor T-cell therapy (CAR-T) has been a promising strategy to achieve long-term remission in relapse leukemias. Unfortunately, those with KMT2A-r have been shown to have a higher risk of CAR-T treatment failure and lineage switch (61). New therapeutic modalities are needed to improve survival and reduce toxicities. As our understanding of the KMT2A and KMT2A-r proteins expands, so has a great interest in therapies targeting KMT2A and/or its protein interactions.

Compared to structural variations, the occurrence and significance of somatic mutations in *KMT2A* in hematologic malignancies are less clear. Few germline *KMT2A* variants appear to be associated with a predisposition to myeloproliferative neoplasms (62, 63). The prognostic and mechanistic role of the somatic and germline *KMT2A* single nucleotide polymorphisms, indels, and cryptic mutations, remains unclear, posing questions of whether KMT2A-targeted therapies are applicable for these KMT2A aberrations.

## Rationally targeting KMT2A-r leukemia

Therapeutic strategies for KMT2A-r leukemia aim to exploit the epigenetic dynamics and the co-occurring aberrant signal transduction (9, 64). The US Food and Drug Administration (FDA) has approved the clinical use of Fms-like tyrosine kinase 3 (FLT3) inhibitors, B cell leukemia/lymphoma 2 (BCL-2) inhibitors, histone deacetylase (HDAC) inhibitors, hypomethylating agents, histone methyltransferase inhibitors, and proteasome inhibitors as targeted cancer therapeutics (65, 66). While these agents have shown reasonable-to-favorable responses in combination with chemotherapeutic agents in KMT2A-r leukemia, they lack the precise targeting of the key oncogenic driver, *i.e.*, KMT2A fusion proteins (65, 66, 67). Recently, there has been a surge in the development of targeted agents directed at the KMT2A fusion proteins, making them highly specific or effective toward malignant cells. Preclinical efforts have suggested that targeting KMT2A fusion-interacting proteins such as DOT1L, pTEFb/CDK9, and BRD4 may be therapeutically valuable (68, 69, 70, 71). The DOT1L inhibitor pinometostat (EPZ-5676) has shown early promise with observable and favorable clinical response as a monotherapy agent in relapse/refractory KMT2A-r disease in adults (NCT01684150) (71). Moreover, the value of pinometostat alongside standard chemotherapy in adult patients with newly diagnosed KMT2A-r AML is under evaluation (NCT03724084). Unfortunately, the same momentum cannot be said for pediatric KMT2A-r, where less than half of patients who received pinometostat monotherapy achieved even transient reduction in leukemic blasts (72). A recent preclinical study however suggests that combination therapy with pinometostat in pediatric leukemia could still be of value (73). Other approaches have included inhibition of pTEFb (positive transcription elongation factor b) and its subunit CDK9 (cyclin-dependent kinase 9). However, the preclinical promise of these inhibitors has not been translated clinically. Atuveciclib (BAY 1143572), a pTEFb inhibitor, and GFH009, a CDK9 inhibitor, have shown limited clinical promise as a monotherapy agent in leukemia (69, 74). Similarly, there are many ongoing efforts to improve bromodomain and extra-terminal (BET) protein inhibitors (75). These compounds have varying affinity for BET proteins BRD2, BRD3, and BRD4, the latter with strong evidence for its involvement in cell cycle progression and cancer metastasis (75). However, the dose escalation study of the BET-inhibitor, RO6870810/TEN-10, showed no therapeutic efficacy in patients with AML (70). Overall, the anticipated benefit of these small-molecule inhibitors has been limited, albeit these trials have been exclusive to patients with relapsed or refractory disease. The therapeutic potential of these inhibitors in *de novo* and newly diagnosed leukemia remains to be investigated.

One class of inhibitors with particular promise blocks the KMT2A-MENIN interaction, MENIN being crucial for the localization of KMT2A and KMT2A fusion proteins to key survival genes in leukemia cells (76). In a Phase I/II clinical trial for the MENIN inhibitor Revumenib (SNDX-5613; NCT04065399), 33% of patients with relapsed or refractory KMT2A-r leukemia achieved complete remission while receiving Revumenib as monotherapy. Of this cohort, most also showed molecular remission *via* flow cytometry and clearance of KMT2A-r in cytogenetic testing (77). Notably, KMT2A fusion targets showed reduced expression, aligning with the preclinical studies *in vivo*. Treatment-related adverse events were tolerable and did not result in the discontinuation of Revumenib (77). Several MENIN inhibitors remain under investigation, as well as their synergistic effects with other targeted agents (76, 78). However, there are emerging concerns about therapeutic resistance and toxicity to these small molecules (79). Recently shown in patient-derived xenografts, the addition of CDK4/6 inhibitor may improve cytotoxicity in MENIN-inhibitor-resistant leukemia cells (80). The current understanding of the KMT2A fusion proteins has inspired many ongoing studies that exploit an expanding list of potential targetable modalities, such as ENL and AF9 YEATS domain (81, 82, 83), as potential new, rational therapeutic targets.

Targeting the WT KMT2A protein in KMT2A-r leukemia is a therapeutic approach also gathering increasing interest. In most KMT2A-associated diseases, one WT *KMT2A* allele is preserved. In KMT2A-r, the WT protein plays an important role in maintaining aberrant gene expression, serving as a pre-requisite for the recruitment of H3K4/K79 methylation (45, 84). The coordination between KMT2A and KMT2A fusion proteins has raised the possibility of therapies targeting WT KMT2A. KMT2A inhibitors can potentially be used to treat other KMT2A aberrant malignancies *e.g.* KMT2A-PTD and KMT2A-a leukemias, which are in desperate need of improved therapies due to their dismal prognosis (42, 85). To target the wild-type function of KMT2A, the initial effort has been to target KMT2A-WDR5 interaction, which is specifically required for the KMT2A methyltransferase activity (86). Preclinical animal models and *in vitro* studies have suggested that peptidomimetic and small molecule inhibitors directed at WDR5 have therapeutic potential for KMT2A-r leukemia (86, 87). Excitingly, selective protein degradation *via* proteolysis targeting chimera (PROTAC) for WDR5 and ASH2L, other core components of the KMT2A complex, also provides a proof-of-concept that such degraders can impact KMT2A-dependent epigenetic landscape and overall gene expression (88, 89). Whether direct degradation of KMT2A is possible, or therapeutically effective, remains to be explored.

## KMT2A aberrations in solid tumor malignancies

As reported in the Catalogue of Somatic Mutations in Cancer (COSMIC), in the order from most to least frequent, nonsense, frameshift, and missense mutations of *KMT2A* are identified in a variety of solid tumor malignancies (5). Conversely, although *KMT2A* structural aberrations have been well characterized in hematological malignancies for the past 3 decades, they have only recently been reported in solid tumors (90, 91, 92). In comparison to hematologic malignancies in which the *KMT2A* N-terminus is preserved, sarcomas with *YAP*::*KMT2A* and *VIM*::*KMT2A* rearrangement have *KMT2A* as the 3′ fusion partner (71). The breakpoints often occur at exons 5 to 6 of the *KMT2A* gene and thus the majority of the KMT2A functional domains, including the CxxC domain and the catalytic SET domain, are preserved in the fusion proteins. Consistently, these sarcoma cells have upregulation of KMT2A canonical targets, that is, *HOX* genes (90, 92). Clinically, the KMT2A-r sarcomas hold poor prognostic value and histologically show infiltrative borders, indicative of their aggressive nature (90). In addition to *YAP*::*KMT2A*, complex rearrangements such as *YAP1*-*KMT2A*-*YAP1* have also been reported in sarcomas, which preserves the CxxC-binding domain, but not the SET domain (90, 91). These sarcomas do not up-regulate *HOX* gene expression (90, 91). Complex and cryptic KMT2A rearrangements suggest multiple underlying mechanisms that remain to be elucidated.

Aside from genetic mutations and structural variations, KMT2A may confer oncogenic function through its interaction proteins. The KMT2A complex can bind to the PHF5A-PHF14-HMG20A-RAI1 protein subcomplex to promote pancreatic cancer progression and serve as a potential therapeutic target (93). KMT2A can also interact with p65 and upregulate cathepsin Z, a known regulator of cancer progression in colorectal cancer (94). *KMT2A* knockdown in colorectal cancer cells decreases cathepsin Z expression as well as tumor invasion both *in vitro* and *in vivo.* (94) The function of KMT2A in solid tumors has only begun to be elucidated. Understanding KMT2A direct downstream targets in different disease contexts may provide new mechanistic and therapeutic insights. The value of KMT2A-directed therapies may be boundless as we discover new functions of KMT2A in gene regulation and disease pathogenesis.

## Conclusion

The KMT2 family stands as a paradigm of epigenetic regulators orchestrating intricate gene expression programs critical for cellular homeostasis and organismal development. Their structural diversity, functional versatility, and regulatory complexity underscore their significance in health and disease. KMT2A has garnered much attention given its well-established presence in syndromes with a neurodevelopmental component, and human malignancy. For leukemias with *KMT2A* aberration, standard treatment regimens rely on chemotherapy and hematopoietic stem cell transplantation, which inherently are burdened with off-target effects and carry a risk of long-term morbidity. Beginning with the landmark contributions of Dr C. David Allis, we have unraveled many of the intricacies of epigenetic regulation which have and will continue to influence innovative approaches to treating adult and childhood cancer. Future efforts on developing effective, mechanism-based therapies against aberrant KMT2A activity and understanding their synergistic potential with conventional drugs will improve the clinical outcome of currently dire diseases.

## Conflict of interest

The authors declare that they have no conflicts of interest with the contents of this article.
